# P-2096. Reverse Blot Hybridization Assay for Simultaneous Multi-drug Resistance Detection of *Helicobacter pylori*

**DOI:** 10.1093/ofid/ofae631.2252

**Published:** 2025-01-29

**Authors:** Kyoung Hwa Lee, Eun Hwa Lee, Sang Hoon Han, Young Goo Song, Hye Young Lee

**Affiliations:** Yonsei University College of Medicine, Seoul, Republic of Korea, Seoul, Seoul-t'ukpyolsi, Republic of Korea; Yonsei University College of Medicine, Seoul, Seoul-t'ukpyolsi, Republic of Korea; Yonsei University College of Medicine, Seoul, Republic of Korea, Seoul, Seoul-t'ukpyolsi, Republic of Korea; Yonsei University College of Medicine, Seoul, Seoul-t'ukpyolsi, Republic of Korea; Yonsei University College of Health Science, Wonju, Kangwon-do, Republic of Korea

## Abstract

**Background:**

*Helicobacter pylori* (*H. pylori*) the eradication is important for cancer prevention. Amoxicillin and clarithromycin, the initial treatments of *H. pylori*, have been shown increasing resistance, suggesting alternative levofloxacin or tetracycline based treatment option. However, theses resistance rate is also increasing by nearly 18-37%, thus, early recognition of multi-drug resistance is critical for efficient treatment success.

**Figure:**
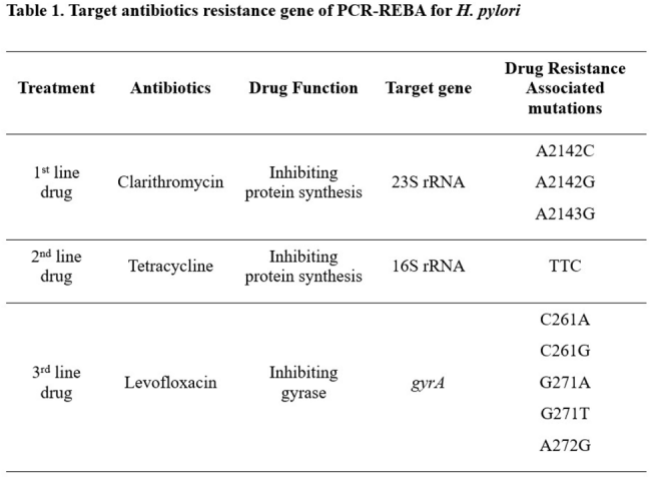

**Methods:**

The novel diagnostic kit is needed that can simultaneously detect multi-drug resistance (Table 1). A total of 311 clinical *H. pylori* isolates were obtained from the gastric endoscopic biopsy in Gangnam Severance Hospital, Seoul, Republic of Korea. The phenotypic drug susceptibility test (pDST) was performed (Table 2), compared with the reverse blot hybridization assay (REBA) tests, and whole genome sequencing was done for the inconsistency.

**Figure:**
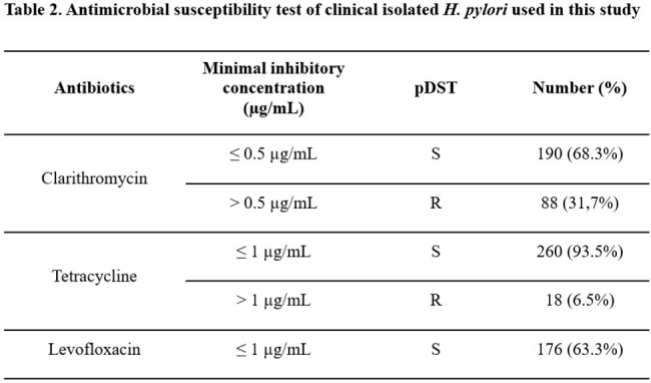

**Results:**

The overall sensitivity about clarithromycin, tetracycline, and levofloxacin are 95.5%, 16.7%, and 55.8%, respectively. The specificity showed much improved detection rate, 96.3%, 92.7%, and 81.8%, respectively (Table 3). Clarithromycin resistance was not significantly different from the performance of the other previously developed kits, and no specific novel mutation sites were discovered other than already known resistance genes. However, novel mutations at A926G, A926T, A926C, A928C, and A934G of tetracycline resistance were discovered. Tetracycline-susceptible clinical *H. pylori* isolates (n = 100) were sequenced at the target region, and these novel mutations were not present. A926G, A926T, A926C showed low-level resistance, with MIC range from 0.36 µg/mL to 1 µg/mL, and the A928C mutation was found in high-level tetracycline resistance (MIC > 2 µg/mL). Among 102 quinolone-resistant *H. pylori* isolates, 45 *H. pylori* isolates showed mismatched with REBA, they had specific sequence (Table 4).

**Figure:**
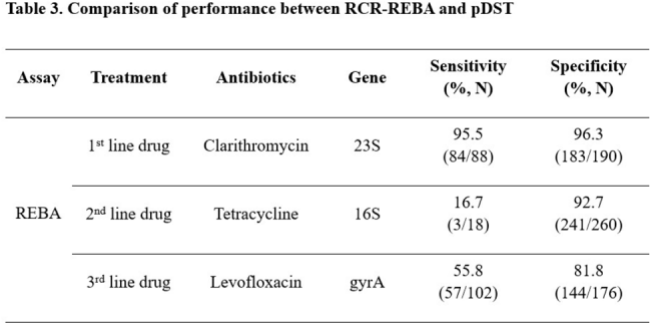

**Conclusion:**

Detection of resistance against *H. pylori* is important in selecting treatment options, therefore, we found novel mutation of tetracycline which can be new target lesion. By simultaneously targeting and detecting various resistance genes, efficiency in antibiotic selection, shortened treatment duration, and reduced medical costs can be expected.

**Figure:**
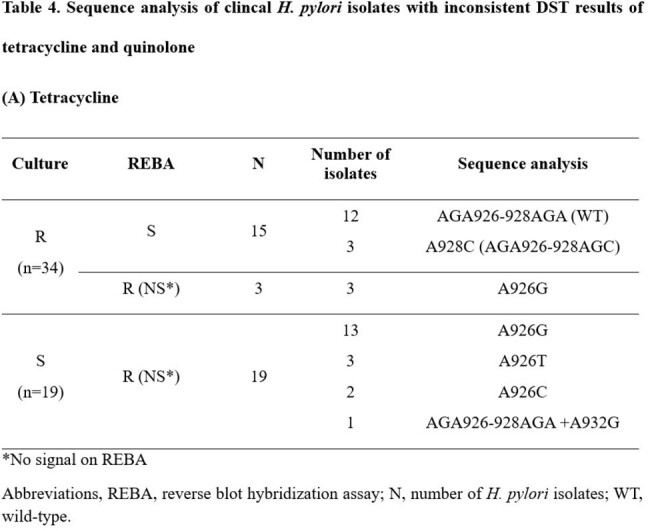

**Disclosures:**

All Authors: No reported disclosures

